# Supplementary material for: Distinct transcriptomic effects of intermittent and chronic caloric restriction in mammary fat pad of a breast cancer mouse model
Source: PLoS One. 2025 Sep 23;20(9):e0331898. doi: 10.1371/journal.pone.0331898 (PMC12456835; doi:10.1371/journal.pone.0331898)
Supplement: S1 File — (PDF) [file pone.0331898.s001.pdf]

1410

Breeding & Maintenance diet for nude rats and mice and transgenic strains

The 1410 formula is a cereal-based (soy, wheat, corn) fixed formula which is free of alfalfa and fish/animal meal and deficient in nitrosamines. This diet was designed as high-energy complete feedingstuff for ad libitum feeding, recommended for nude/transgenic rodents in growing period, breeding and maintenance.

Available as

1414 - 10mm pellets

Product variations

The 1410 formula is optionally also available in one or a combination of the following variants.

Variant P
deficient in phytoestrogens
Variant Fortified
autoclavable, increased vitamin content, coated on request
Variant Irradiated
irradiated with 5 to 50kGy

The contents may vary for these variants.

Metabolized energy

Content	Value		unit
Fat	821	(22%)	kcal/kg
Protein	1,013	(28%)	kcal/kg
Carbonhydrates	1,845	(50%)	kcal/kg

crude nutrients and moisture

Content	Value		unit
Moisture	100,581	(10.1%)	mg/kg
Crude Ash	57,079	(5.7%)	mg/kg
Crude Fibre	24,490	(2.4%)	mg/kg
Crude Fat	91,261	(9.1%)	mg/kg
Crude Protein	253,253	(25.3%)	mg/kg
Nitrogenfree extractives	473,336	(47.4%)	mg/kg

Altromin Spezialfutter GmbH & Co. KG
Im Seelenkamp 20, 32791 Lage, Germany
info@altromin.de - www.altromin.com

Carbonhydrates

Content	Value	unit
Monosaccharides	23,750	mg/kg
Disaccharides	51,115	mg/kg
Polysaccharides	304,628	mg/kg

Minerals

Content	Value	unit
Calcium	7,026	mg/kg
Potassium	8,714	mg/kg
Magnesium	1,822	mg/kg
Sodium	2,170	mg/kg
Phosphorus	5,111	mg/kg

Trace elements

Content	Value	unit
Aluminium	81.16	mg/kg
Chlorine	3,365.80	mg/kg
Iron	182.35	mg/kg
Flourine	3.44	mg/kg
Iodine	1.49	mg/kg
Cobalt	0.36	mg/kg
Copper	13.67	mg/kg
Manganese	72.41	mg/kg
Molybdenum	1.28	mg/kg
Sulfur	864.20	mg/kg
Selenium	0.24	mg/kg
Zinc	79.04	mg/kg

Altromin Spezialfutter GmbH & Co. KG
Im Seelenkamp 20, 32791 Lage, Germany
info@altromin.de - www.altromin.com

Added vitamins

Content	Standard	Fortified	unit
Vitamin A	15,000	26,250	IU/kg
Vitamin D3	600	1,050	IU/kg
Vitamin E	81	138	mg/kg
Vitamin K3	3	5	mg/kg
Vitamin B1	18	32	mg/kg
Vitamin B2	12	21	mg/kg
Vitamin B6	9	16	mg/kg
Vitamin B12	24	42	µg/kg
Nicotinic acid	36	63	mg/kg
Pantothenic acid	21	37	mg/kg
Folic acid	2	4	mg/kg
Biotin	230	275	µg/kg
Choline chloride	600	1,050	mg/kg
Vitamin C	36	63	mg/kg

Amino acids

Content	Value	unit
Alanine	13,811	mg/kg
Arginine	14,895	mg/kg
Aspartic acid	22,607	mg/kg
Cystine	3,970	mg/kg
Glutaminc acid	40,497	mg/kg
Glycine	10,824	mg/kg
Histidine	6,257	mg/kg
Isoleucine	10,965	mg/kg
Leucine	15,583	mg/kg
Lysine	11,852	mg/kg
Methionine	4,343	mg/kg
Phenylalanine	13,142	mg/kg
Proline	19,510	mg/kg
Serine	13,259	mg/kg
Threonine	9,619	mg/kg
Tryptophan	2,861	mg/kg
Tyrosine	10,200	mg/kg
Valine	12,564	mg/kg

Altromin Spezialfutter GmbH & Co. KG
Im Seelenkamp 20, 32791 Lage, Germany
info@altromin.de - www.altromin.com

Fatty acid

Content	Value	unit
Arachidic acid C-20:0	456	mg/kg
Eicosanoic acid C-20:1	570	mg/kg
Alpha-Linolenic acid C-18:3	5,720	mg/kg
Linolenic acid C-18:2	41,619	mg/kg
Palmitic acid C-16:0	10,215	mg/kg
Stearic acid C-18:0	3,055	mg/kg
Oleic acid C-18:1	17,568	mg/kg

Altromin Spezialfutter GmbH & Co. KG
Im Seelenkamp 20, 32791 Lage, Germany
info@altromin.de - www.altromin.com
