# Supplementary material for: Distinct transcriptomic effects of intermittent and chronic caloric restriction in mammary fat pad of a breast cancer mouse model
Source: PLoS One. 2025 Sep 23;20(9):e0331898. doi: 10.1371/journal.pone.0331898 (PMC12456835; doi:10.1371/journal.pone.0331898)
Supplement: S1 Table — (DOCX) [file pone.0331898.s002.docx]

**S1 Table:** Number of the MFP tissue samples for each dietary group at each time point of the study.

| **Group** | **Baseline, Week 10** | **Week 49/50** | **Week 81/82** |
| --- | --- | --- | --- |
| AL | 5 | 5 | 4 |
| CCR | - | 4 | 5 |
| ICR-R | - | 5 | 5 |
| ICR-RF | - | 4 | 5 |
