# Supplementary figures and images for: Distinct transcriptomic effects of intermittent and chronic caloric restriction in mammary fat pad of a breast cancer mouse model
Source: PLoS One. 2025 Sep 23;20(9):e0331898. doi: 10.1371/journal.pone.0331898 (PMC12456835; doi:10.1371/journal.pone.0331898)

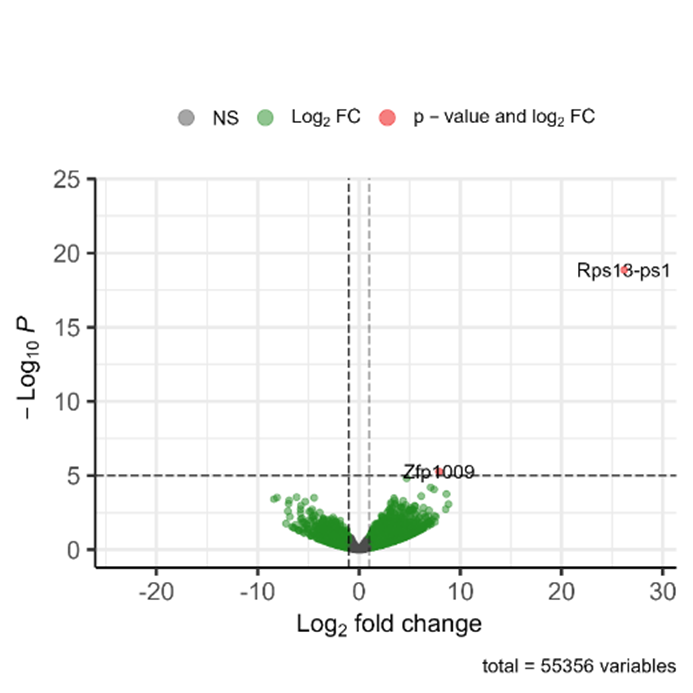

Supplement: S1 Fig — Volcano graph displays the differentially expressed genes (DEGs) in the ICR-RF group compared to the ICR-R group at adult age. Absolute log2 fold-change value of >2 and adjusted p value of 0.1 were considered statistically significant. (TIF) [file pone.0331898.s003.tif]

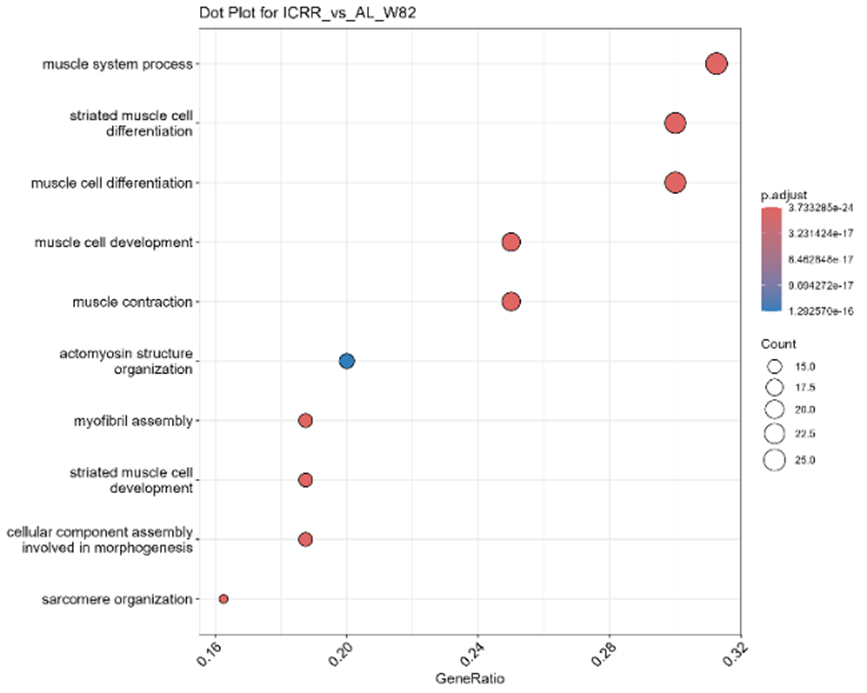

Supplement: S2 Fig — Dot plot represents the enriched pathways of the upregulated genes in the ICR-R group compared to the AL group at old age. Adjusted p value of 0.1 were considered statistically significant. (TIF) [file pone.0331898.s004.tif]

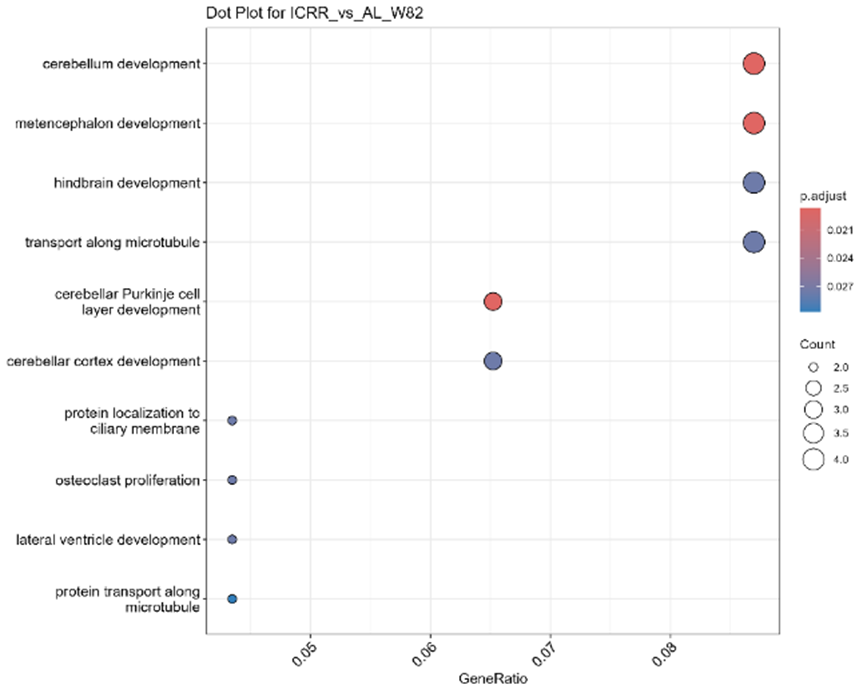

Supplement: S3 Fig — Dot plot represents the enriched pathways of the downregulated genes in the ICR-R group compared to the AL group at old age. Adjusted p value of 0.1 were considered statistically significant. (TIF) [file pone.0331898.s005.tif]

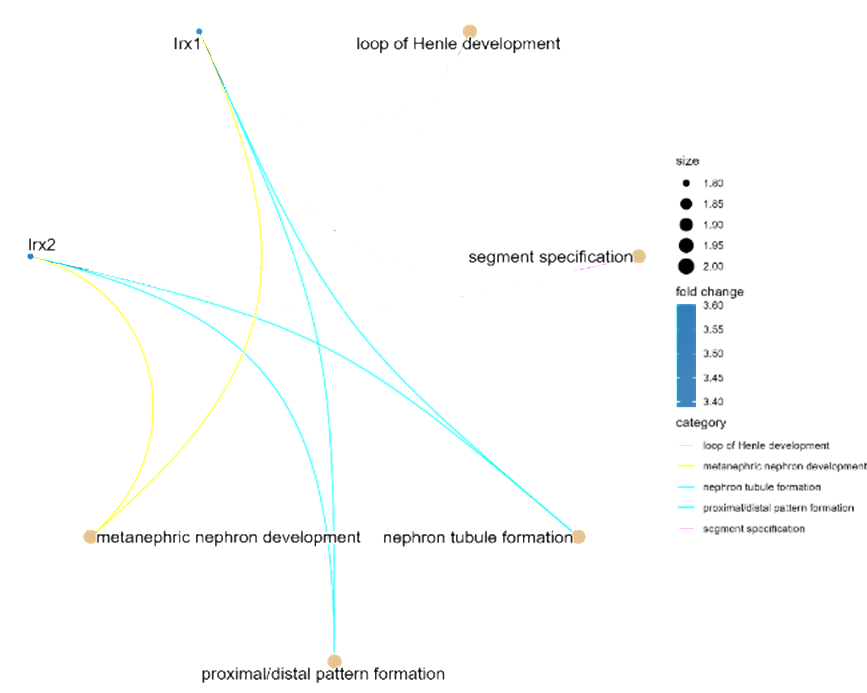

Supplement: S4 Fig — Circular network plot displays the relationship between the DEGs and the enriched pathways in the ICR-RF group compared to the ICR-R group at old age, with fold-change values. Absolute log2 fold-change value of >2 and adjusted p value of 0.1 were considered statistically significant. (TIF) [file pone.0331898.s006.tif]
